# Effect of Coupled Wing Motion on the Aerodynamic Performance during Different Flight Stages of Pigeon

**DOI:** 10.34133/cbsystems.0200

**Published:** 2025-03-11

**Authors:** Yishi Shen, Yi Xu, Weimin Huang, Chengrui Shang, Qing Shi

**Affiliations:** ^1^ Intelligent Robotics Institute, School of Mechatronical Engineering, Beijing Institute of Technology, Beijing 100081, China.; ^2^ Key Laboratory of Biomimetic Robots and Systems, Beijing Institute of Technology, Ministry of Education, Beijing 100081, China.; ^3^ Key Laboratory of Animal Ecology and Conservation Biology, Institute of Zoology, Chinese Academy of Sciences, Beijing 100101, China.; ^4^ University of Chinese Academy of Sciences, Beijing 100049, China.; ^5^ Yangtze Delta Region Academy of Beijing Institute of Technology, Jiaxing 314000, China.

## Abstract

Birds achieve remarkable flight performance by flexibly morphing their wings during different flight stages. However, due to the lack of experimental data on the free morphing of wings and the complexity of coupled motion in aerodynamics studies, the intricate kinematic changes and aerodynamic mechanisms of wings during various flight stages still need to be explored. To address this issue, we collected comprehensive data on free-flight pigeons (*Columba livia*). We categorized the wing kinematic parameters during the takeoff, leveling flight, and landing stages into 5 kinematics parameters: flap, twist, sweep, fold, and bend. Based on this, we established a 3-dimensional pigeon wing model, defined its coupled motion using rotation matrices, and then used the computational fluid dynamics method to simulate the coupled motion in the 3 flight stages. We analyzed and compared the kinematic parameter changes, aerodynamic forces, and flow structures. It is found that, within a wingbeat cycle, pigeons during the takeoff stage cause the leading-edge vortex to attach earlier, enhancing instantaneous lift to overcome gravity and achieve ascending. During the leveling flight stage, the pigeon’s average lift becomes stable, ensuring a steady flight posture. In the landing stage, the pigeon increases the wing area facing the airflow to maintain a stable landing posture, achieving a more minor, consistent average lift while increasing drag. This study enhances our understanding of birds’ flight mechanisms and provides theoretical guidance for developing efficient bio-inspired flapping-wing aerial vehicles.

## Introduction

In nature, birds attract attention for their extraordinary flight capabilities, taking off, maneuvering in flight, and landing autonomously [1–3]. During the entire flight process, birds attain precise flight control by adaptively morphing the shape of their wings across various flight stages [4–6]. In contrast, existing flapping-wing aerial vehicles (FWAVs) struggle to achieve the agility of birds. Therefore, understanding the intricate kinematic changes of wings at different flight stages and their aerodynamic mechanisms is crucial for comprehending bird flight functions. It also provides inspiration and insights for the design of FWAVs.

Existing descriptions of wing kinematics often express the complex wing movements as flapping, angle of attack (AOA) changes due to twisting, or folds caused by wing contraction [5–7]. Moreover, previous studies have used high-speed cameras to collect wing morphing on hovering hummingbirds. The hovering motion was divided through wing reconstruction into spanwise bending, camber, and spanwise twists [8]. Unlike hummingbirds, medium-sized birds like pigeons and owls require more free-flight space, and critical wing positions may be obscured during movement. Tobalske et al. [9] established a long flight corridor and detailed the kinematic parameters of wing movements during slide, pitch, and plunge phases through a 3-dimensional (3D) reconstruction of the wing surface. Berg and Biewener [10] collected flight data of pigeons, dividing their wings into 3 planes and describing the average angular changes for each plane. Similarly, Durston et al. [11] conducted experiments on 3 different bird species to investigate whether wing deformation affects flight but only examined changes in the distal wing and body angle. Wolf and Konrath [12] reconstructed the barn owl wing by a similar method description of the spanwise varying kinematics and aerodynamic parameters. In the kinematic study of pigeons, Ju et al. [13] built a 10 m × 10 m × 7 m space and used a motion capture system to collect data on freely flying pigeons, but only analyzed the degrees of freedom (DOFs) of the shoulder, elbow, and wrist joints. Nevertheless, there is still a lack of detailed descriptions of the complete wing kinematics of birds with outstanding flight performance. Pigeons, known for their excellent flight performance, complex wing movements, and long history of domestication, were chosen as the experimental subjects in this study.

To analyze the effects of changes in wing shape on the aerodynamic performance of birds in flight, wind tunnel experiments are often used [14,15]. Despite that, due to the limitations of the scenarios, wind tunnels designed to study aerodynamic performance cannot meet the requirements for capturing the entire process of free flight simultaneously. Existing studies can reconstruct birds’ wing and body surface structures through scanning and use computational fluid dynamics (CFD) simulations to analyze the aerodynamic performance during flight [16,17]. By reconstructing the overall geometric model of a goose and using a dual-joint wing model with NACA airfoils, analysis of wingtip vortices with an up-and-down flapping model indicates that migratory birds seem to use ascending areas during migration to increase lift and save energy costs [18]. Similarly, Huang et al. [19] used bird model reconstruction methods, and CFD was employed to study the numerical values of the webbed feet, body, feathers around the wings, and hydrodynamics during a cormorant’s takeoff from the water, validating the power provided by the webbed feet during the takeoff phase. Beratlis et al. [20] captured the motion of a horned owl using multiple cameras, conducted high-precision reconstruction, configured the wing model’s movements, and performed aerodynamic simulations of the flight process. However, in these studies, actual bird movements are often simplified, and the aerodynamic analysis of coupled wing motion is also not the main focus.

Although there have been some studies that have defined the coupled motion of the wings. Studies of Bie et al. and Lang et al. [21,22] have performed simulation analyses of flapping couplings within the stroke plane, investigating the impact of different flapping angles on aerodynamic performance for both inner and outer wings. Chang et al. introduced fold into the flapping motion to examine its effect on drag coefficients. Additionally, 2-parameter couplings have been explored to enhance lift, with a study incorporating bend angles in bat wing simulations and further investigating the impact of adding sweep angles on wing performance [23]. Another study analyzed 3 types of motion simulations: plunging within the stroke plane, twisting along the wingspan, and sweeping outside the stroke plane, studying the effects of both individual and coupled motions on lift enhancement through alula [24]. However, these definitions of coupled motion are engineering-based and do not accurately reflect the deformation occurring within a bird’s wingbeat. Thus, applying accurate bird motion data to CFD simulations is essential for accurate research.

In this paper, we propose a CFD simulation method based on biological experimental data to analyze the aerodynamic performance of pigeons during takeoff, leveling flight, and landing in free flight. First, we used 30 motion capture cameras in a 16 m × 5 m × 3 m space to collect the wing movement data of pigeons throughout the entire free flight process. Secondly, we decoupled and analyzed the complex coupled wing movements into 5 kinematic parameters: flapping, twisting, sweeping, folding, and bending. A wing model was constructed to conduct aerodynamic simulations by simplifying the scanned wing surface profiles. The wing’s movements were defined using rotation matrices employed in the simulation process. To better understand the aerodynamic mechanisms of the wings during 3 different stages, we used CFD methods to analyze the aerodynamic characteristics of the coupled movements of the 5 kinematic parameters. Furthermore, we provided a detailed analysis of the flow field structures during each process. To our best knowledge, this study is the first to conduct a CFD coupled-motion analysis across all flight stages using biological data, revealing the aerodynamic characteristics of medium-sized birds at different stages of flight. This study will improve our understanding of the coupled movements during the flight process of natural fliers and provide theoretical guidance and inspiration for developing bio-inspired FWAVs.

## Materials and Methods

### Data collection and simplified pigeon wings

This study mainly aims to investigate pigeon wings’ aerodynamic characteristics and kinematic parameters during takeoff, leveling flight, and landing. Due to the difficulty of collecting data from live pigeons, quantifying specific wing kinematic parameters becomes challenging. Current research seldom includes aerodynamic simulations of live pigeons in various flight states. To obtain kinematic data of live pigeons during various stages of free flight, we established a free-flight space with dimensions of 16 m × 5 m × 3 m. This space was set up indoors in a windless environment, with no obstructions along the pigeon’s flight path. Therefore, to ensure the stability of the data, the pigeons will not have an emergency takeoff or landing status. Data were collected using 30 Optitrack Prime13 motion capture cameras operating at a sampling frequency of 240 Hz. The cameras were calibrated using a 500-mm calibration wand, resulting in an average wand error of 0.134 mm and a residual error of 0.392 mm, ensuring precise data collection.

**Fig. 1.**
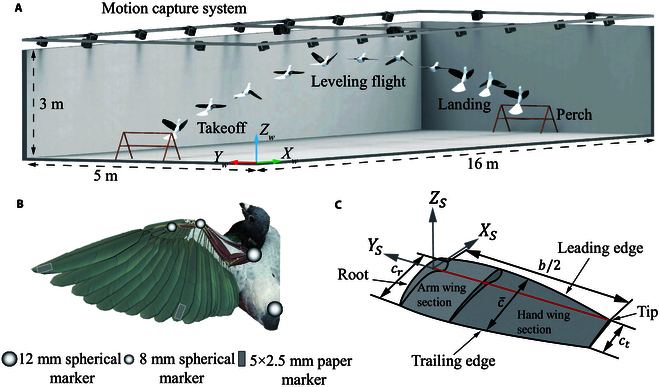
Overview of the pigeon flight experiment and wing model. (A) The flight experiment scene consists of a 16 m ×5m× 3 m space, with 30 motion capture cameras set up. The pigeon takes off from a perch at one end and lands at the other. (B) Markers on the pigeon’s body and wings include 12-mm spherical markers, 8-mm spherical markers, and 5-mm × 2.5-mm paper markers. (C) 3D wing model, representing the right wing of the pigeon.

To guarantee that no external factors are interfering with the flight, the indoor lighting was white, designed to avoid stimulating the pigeons, and the camera exposure was set to 50 μs. The detailed experimental setup is illustrated in Fig. 1A. Before formal data collection, we trained each pigeon for 30 min daily to acclimate them to the experimental setup and encourage them to fly between 2 perches. Eventually, the pigeons could voluntarily take off, engage in consecutive free flights, and land at a designated target point, all without human interference. The experiment collected free-flight data from 5 adult homing pigeons (*Columba livia*, 2 to 3 years old). Each pigeon undergoes more than 20 times complete flight data collection from takeoff to landing. After data labeling and filtering, 5 flight datasets for each pigeon were selected for analysis. In this study, we only presented the average angles of each pigeon.

To obtain accurate kinematic data of the wings. The reflective markers on the wings were placed at the pigeon’s shoulder joint, wrist joint, metacarpal joint, wingtip, junction of primary and secondary feathers, and wing root. Fig. 1B shows the pigeon’s wings and body markers. The positions of the skeletal points were determined by computed tomography (CT) scanning, and the body markers were placed on the pelvis to facilitate the establishment of the body coordinate system during analysis. Reflective markers on the wings were used to capture detailed kinematic changes, whereas body markers were used to compute the pigeon’s transformation relative to the world coordinate system. We used 3 reflective markers to avoid affecting the pigeon’s flight while ensuring the motion capture system could collect as many markers as possible. The 12-mm larger reflective markers were attached to the pigeon’s shoulder joints and rump, 8-mm markers were attached to the wrist joints and metacarpals, and trimmed, lighter paper markers were used on the feathers.

The airfoil data of the wings were determined based on the CT scan results of a fully extended homing pigeon as referenced in the paper [16]. The paper divided the wing scan cross-sections into 9 different airfoil segments. In this study, the airfoils were simplified, and only the airfoils near the anatomical wrist joint, wing root, and wingtip were selected for wing model construction.

### Wingbeat kinematics parameters

During the flight, the pigeon’s various movements can obscure the markers attached to the wings, preventing the motion capture cameras from accurately detecting them. Therefore, we performed cubic spline interpolation and data smoothing on the collected points to guarantee data validity. Additionally, by filtering the relative positions of the wingtip and shoulder joint, we identified the 3 different flight stages: takeoff, leveling flight, and landing. The spanwise length of the arm wing section is approximately $l_{1}=0.115\;m$.

To better represent the wingbeat kinematics of a pigeon during flight, the wing model was divided into the hand wing and arm wing sections at the metacarpal joint position, as shown in Fig. 1C. The $O_{S}-x_{S}y_{S}z_{S}$ coordinate system represents a coordinate system centered at the pigeon’s shoulder joint, with the spanwise direction of the simulation model being the negative *Y* direction. Here, $c_{r}$ represents the chord length at the wing root, and $c_{t}$ represents the chord length at the wingtip. The mean chord length $\bar{c}=0.125\;m$, the wingspan is denoted as *b*, and the half-span used for simulation calculations is b/2=0.2406m.

**Fig. 2.**
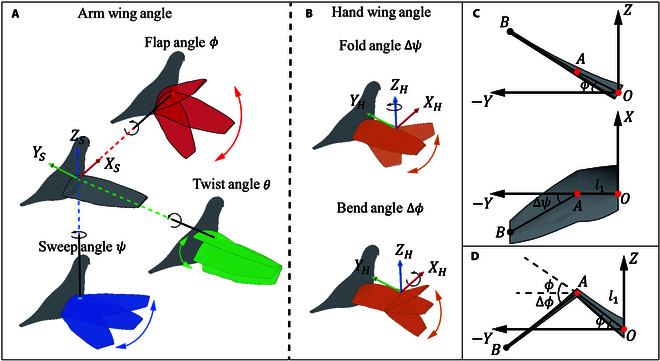
(A) Detailed description of arm wing and hand wing angles: the arm wing includes flap angle ϕ, twist angle θ, and sweep angle ψ. (B) The hand wing includes fold angle Δψ and bend angle Δϕ. (C) Coordinate representation of flap and bend movements, where $l_{1}$ represents the length of the arm wing, and point *A* represents the rotation center of the hand wing. (D) Coordinate representation of fold movement, divided into flapping in the *ZY* plane and folding in the *XY* plane.

To comprehensively characterize wing kinematics, we decomposed the motion into the $O_{s}-x_{s}y_{s}z_{s}$ coordinate system centered at the shoulder joint and the $O_{H}-x_{H}y_{H}z_{H}$ coordinate system centered at the metacarpal joint, representing 5 kinematic parameters of one wingbeat cycle. Fig. 2A shows the $O_{s}-x_{s}y_{s}z_{s}$ coordinate system; there is the flapping angle ϕ rotating around the $X_{S}$ axis, the twisting angle θ rotating around the $Y_{S}$ axis, and the sweeping angle ψ rotating around the $Z_{S}$ axis. These 3 movements represent the kinematic parameters of the arm wing section. Fig. 2B shows the $O_{H}-x_{H}y_{H}z_{H}$ coordinate system; the motions are divided into the folding angle Δψ of the hand wing rotating around the $Z_{H}$ axis and the bending angle Δϕ rotating around the $X_{H}$ axis. The coupling of these 5 kinematics parameters constitutes the wingbeat motion of a pigeon.

Although we divided the wingbeat kinematics individually, in subsequent CFD simulations, these movements are based on the coupled motion of the flap. The coupled motion can be obtained through multiple coordinate transformations. Due to the substantial differences in motion between the hand wing and the arm wing in the metacarpal coordinate system, it is necessary to analyze their coordinate transformations. In the inertial coordinate system, the arm wing coordinates are represented by $P_{\mathrm{arm}}=\;{\left(x_{s},\;y_{s},\;z_{s}\right)}^{T}$. $P_{\text{hand}}=\;{\left(x_{h},\;y_{h},\;z_{h}\right)}^{T}$ represents the hand wing coordinates. Fig. 2C shows the bend-coupled motion in the *ZY* plane. The arm wing section has a length of $l_{1}$ and rotates around the origin *O* with an angle of ϕ, forming the flap motion. The hand wing section rotates around point *A*, which is the wrist joint of the pigeon, forming the bend motion. Therefore, the coupling of the bend motion in Fig. 2C can be represented by the translation and rotation matrices of the flap angle and the bend angle of the hand wing section:

$$\begin{array}{c} \left[\begin{array}{c} P_{\mathrm{arm}}^{\prime }\\ 1 \end{array}\right]=R_{1}\left[\begin{array}{c} P_{\mathrm{arm}}\\ 1 \end{array}\right],\left[\begin{array}{c} P_{\text{hand}}^{\prime }\\ 1 \end{array}\right]=T_{2}R_{2}T_{1}R_{1}\left[\begin{array}{c} P_{\text{hand}}\\ 1 \end{array}\right]\; \end{array}$$

The rotation matrix $R_{1}$, $R_{2}$ and translation matrix $T_{1}$, $T_{2}$ are represented as follows:

$$\begin{array}{c} \begin{array}{cc} R_{1}=\left[\begin{array}{cccc} 1 & 0 & 0 & 0\\ 0 & \text{cos}\phi & -\text{sin}\phi & 0\\ 0 & \text{sin}\phi & \text{cos}\phi & 0\\ 0 & 0 & 0 & 1 \end{array}\right]\;\;\;\;\;T_{1}=\left[\begin{array}{cccc} 1 & 0 & 0 & 0\\ 0 & 1 & 0 & -y_{A}\\ 0 & 0 & 1 & -z_{A}\\ 0 & 0 & 0 & 1 \end{array}\right] & \\ R_{2}=\left[\begin{array}{cccc} 1 & 0 & 0 & 0\\ 0 & \text{cos}\Delta \phi & -\text{sin}\Delta \phi & 0\\ 0 & \text{sin}\Delta \phi & \text{cos}\Delta \phi & 0\\ 0 & 0 & 0 & 1 \end{array}\right]\;\;\;\;T_{2}=\left[\begin{array}{cccc} 1 & 0 & 0 & 0\\ 0 & 1 & 0 & y_{A}\\ 0 & 0 & 1 & z_{A}\\ 0 & 0 & 0 & 1 \end{array}\right] & \end{array} \end{array}$$ (1)

Here, $y_{A}=l_{1}\text{cos}\phi$ and $z_{A}=l_{1}\text{sin}\phi$ represent the positional transformations of the wrist joint at point *A*, respectively. The positional transformation of the hand wing section can be obtained by calculating the flap and the bend angle.

The difference between the coupled and bending motion is that folding involves the wing’s movement in the *XY* plane. Fig. 2D shows that the fold motion is first analyzed in the coordinate transformation. Then, the whole wing is analyzed using the *ZY* plane. Hence, it is represented by a rotation matrix:

$$\begin{array}{c} \begin{array}{cc} P_{\text{fold}}=P_{\mathrm{arm}}+P_{\text{hand}}, & \\ \left[\begin{array}{c} P_{\text{flap}}^{\prime }\\ 1 \end{array}\right]=R_{1}\left[\begin{array}{c} P_{\text{fold}}\\ 1 \end{array}\right],\left[\begin{array}{c} P_{\text{hand}}^{\prime }\\ 1 \end{array}\right]=R_{3}\left[\begin{array}{c} P_{\text{hand}}\\ 1 \end{array}\right] & \end{array} \end{array}$$ (2)

The rotation matrix $R_{3}$ is represented as follows:

$$R_{3}=\left[\begin{array}{cccc} \text{cos}\Delta \psi & -\text{sin}\Delta \psi & 0 & 0\\ \text{sin}\Delta \psi & \text{cos}\Delta \psi & 0 & 0\\ 0 & 0 & 0 & 0\\ 0 & 0 & 0 & 1 \end{array}\right]$$ (3)

Unlike bend and fold motions involving different kinematic patterns of the arm and hand wings, twist and sweep motions can be directly obtained by 2 overall wing coordinate rotations.

### Mesh and computational methods

This study employs the commercial CFD software Fluent to solve the transient simulation process of the pigeon’s right wing in the takeoff, leveling flight, and landing stages. Since this paper has already detailed the division of all motion angles on the wings, covering all deformations within a wingbeat cycle, including those caused by air pressure-induced feather deformation, we only consider the simulation of a rigid wing model in this study. Moreover, the entire process does not consider the effect of the body AOA on wing motion. The equations governing the flow in the numerical solver are the transient and incompressible continuity. All the coupled wing motions are implemented through user-defined functions (UDFs). To achieve complex motions, the DEFINE_GRID_MOTION macro is used to define the grid changes of the wing. Due to the involvement of complex wing motions, the N_UDMI function is also used during the motion definition process. The initial position of the wing grid is horizontal in the $O_{w}-x_{w}y_{w}$ plane, so its initial position is stored in the FLUENT memory by using the N_UDMI function. The N_UDMI function can store the initial position’s *X*, *Y*, *Z* position information. The rotation matrix in the “Wingbeat kinematics parameters” section calculates the grid position in the next step of the dynamic mesh.

In the dynamic mesh settings, the motion of the grid around the wing is defined using the smoothing method. Smoothing is often used to smooth grid node positions and helps improve the mesh quality in deformed areas. It reduces numerical errors caused by highly distorted meshes. The simulation of wing motion is solved by solving the unsteady Reynolds-averaged Navier-Stokes equations. The solver in Fluent is chosen as the k−ω-based shear stress transport (SST) turbulence model. This model is often used for low Reynolds number simulations. The SIMPLEC algorithm is chosen for pressure-velocity coupling calculations in the solution control methods. For spatial discretization, second order is used for pressure. Second-order upwind methods are selected for momentum, turbulent kinetic energy, and specific dissipation rate.

**Fig. 3.**
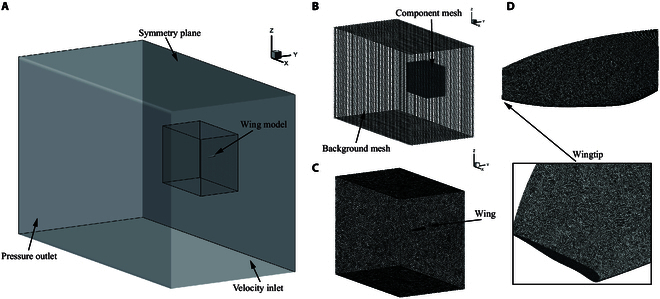
Computational domain for pigeon wing. (A) Overview of the computational field. The velocity inlet and pressure outlet. (B) Mesh composition, with an outer Cartesian mesh and an inner unstructured mesh. (C) The inner unstructured mesh is refined to guarantee the mesh quality around the wing. (D) The wing’s surface mesh is generated using an unstructured mesh. Detailed illustration of the wingtip mesh.

The computational domain for transient simulation is shown in Fig. 3A. The computational domain comprises an outer stationary region and an inner deforming region. The global dimensions of the computational domain are 80c × 40c × 50c. The size of the deforming region is 16c × 12c × 20c. The specific grid division is shown in Fig. 3B. The outer background region grid is composed of Cartesian grids. Fig. 3C shows the inner region comprising refined unstructured grids. The wing surface is also meshed with unstructured grids.

The Reynolds number is determined by the different flight speeds in the 3 stages. The Reynolds number is expressed as:

$$R_{e}=\frac{\rho \mathrm{Ub}}{2\mu }$$ (4)

where ρ represents the air density, *U* is the speed at different flight stages, *b* is the wingspan, and μ is the dynamic viscosity of the fluid. The Reynolds number for the takeoff stage is $R_{e_{\text{takeoff}}}=18,950$, that for the flight stage is $R_{e_{\text{flight}}}=41,257$, and that for the landing stage is $R_{e_{\text{land}}}=29,724$.

The wing’s lift, drag, and pressure are obtained and analyzed through simulation. The dimensionless moment coefficient is expressed by:

$$\begin{array}{cc} C_{L}=L/\left(\frac{1}{2}\rho U^{2}S\right), & \\ C_{D}=D/\left(\frac{1}{2}\rho U^{2}S\right), & \\ C_{T}=-C_{D}, & \\ C_{P}=\left(p-p_{\infty }\right)/\left(\frac{1}{2}\rho U^{2}S\right) & \end{array}$$ (5)

where *L* represents lift, the force perpendicular to the flight velocity direction, *D* represents drag, the force parallel to the flight direction, *S* represents the wing area when fully extended, *p* represents the calculated static pressure, and $p_{\infty }$ represents the pressure at infinity.

### Solver validation

We validated the chosen solver in this CFD simulation according to the methodology in Ref. [22]. The validation experiment used a NACA0012 cross-section airfoil with a wing span of 600 mm and a chord length of 100 mm. The wing model was selected as in Ref. [25] for the flapping wing solver validation simulation. The displacement of the wing root *S* is expressed as:

$$S=h_{\text{Base}}\text{cos}\left(2\pi \;\mathrm{ft}\right)$$ (6)

where $h_{\text{Base}}=0.175c$, and the phase difference between wing tip and wing root is 0. The Reynolds number is 30,000, reduced frequency is defined as $k=\pi \;\mathrm{fc}/U_{o}=1.82$, and $U_{o}$ is the incoming flow velocity equal to 0.393m/s.

**Fig. 4.**
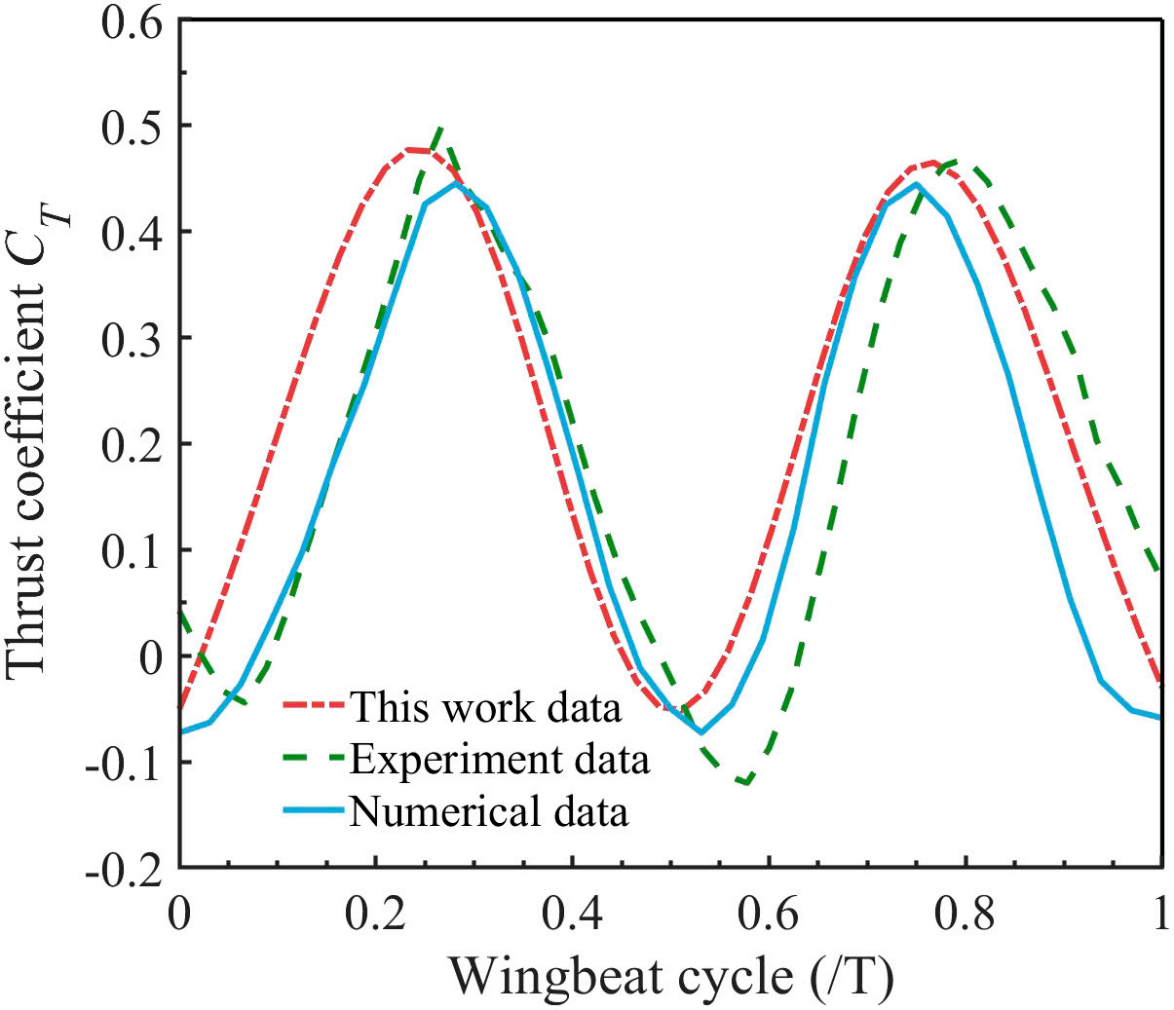
Validation of the thrust coefficients. The red dashed line represents thrust coefficients from the present simulation. The green dashed line represents experiment data from Ref. [25] and the blue line represents numerical data from Ref. [26].

To validate our simulations, the same meshing method in the previous subsection was chosen, the computational domain is 80c × 40c × 50c, the motion of the wing is controlled by the DEFINE_GRID_MOTION macro of the UDF, the time step is also set to T/200, and the simulation was run for 5 consecutive cycles. Fig. 4 shows the thrust coefficient in the last cycle of the 5 consecutive cycles compared to Refs. [25,26]. It can be concluded from Fig. 4 that the results of the present simulation have similar results to the previous work. Specifically, there is a trend toward similarity with experimental data.

### Mesh and time step independence studies

**Fig. 5.**
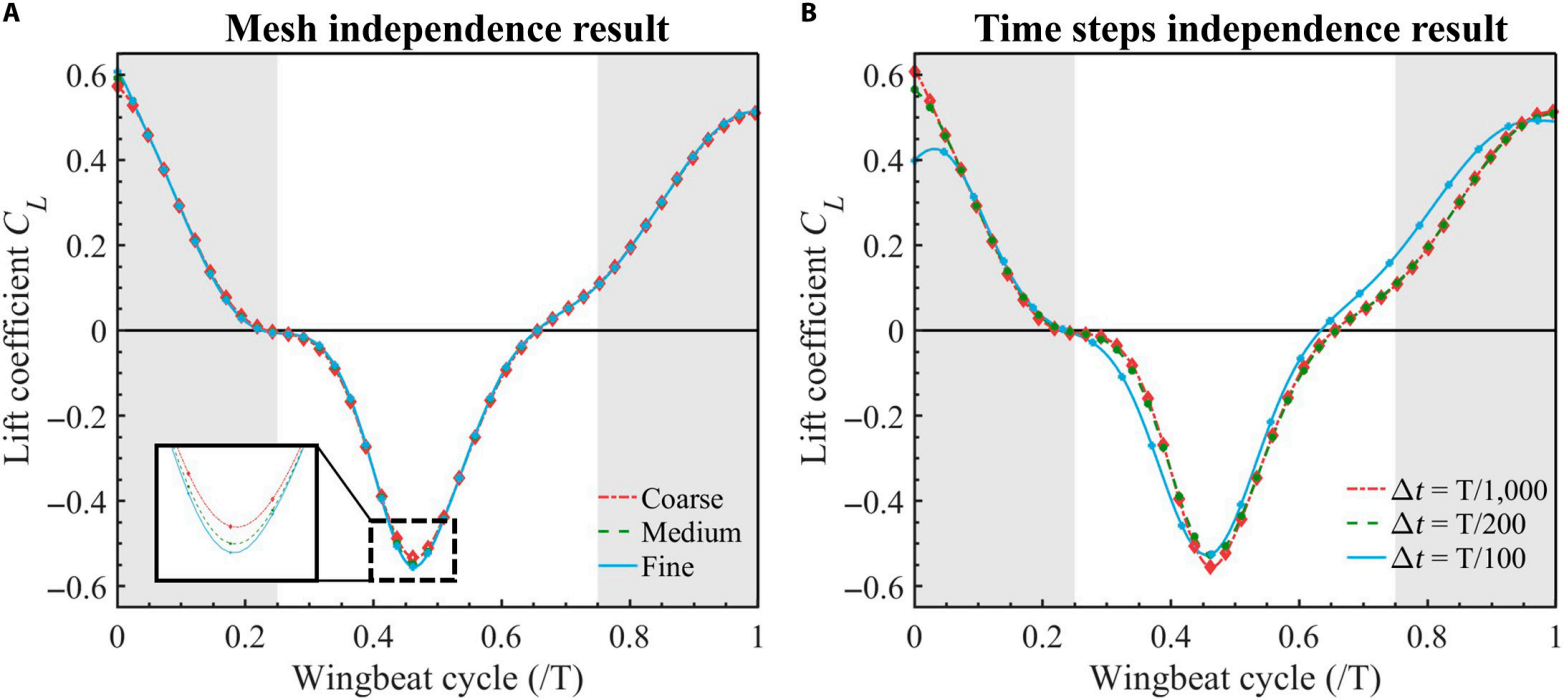
Validation of the thrust coefficients. (A) Mesh independence test results. The red dotted line refers to Coarse mesh. The green dot line refers to Medium mesh. The blue mesh refers to Fine mesh. (B) Time step independence test results. The red dotted line refers to T/10,001. The green dotted line refers to T/200. The blue mesh refers to T/100.

To verify the mesh resolution and time step effect on the solution results. We use the flapping motion during the leveling flight phase to define the pigeon wing motion and divide the mesh resolution and time steps into 3 types. The grid resolution is divided into coarse ($8\times 10^{6}$), medium ($3.6\times 10^{7}$), and fine ($1.4\times 10^{8}$). The results are shown in Fig. 5A. Small differences can be seen in the 3 mesh resolutions. The 3 mesh resolutions subtly only in the low peaks in the middle of the upstroke. Therefore, taking into account the computational power and the stability of the results, we finally chose the medium mesh for the calculation.

Based on the medium mesh, the time steps are divided into T/100, T/200, and T/1,000. The results are shown in Fig. 5B. Unlike the mesh independence results, the time step has a greater effect on the results. It can be found in Fig. 5B that the results of T/100 are clearly different from T/200 and T/1,000. However, there is no significant difference between T/200 and T/1,000, while there is a 5-times difference in the time step. Therefore, the time step is chosen as T/200.

## Results

### Wingbeat kinematics analysis

**Fig. 6.**
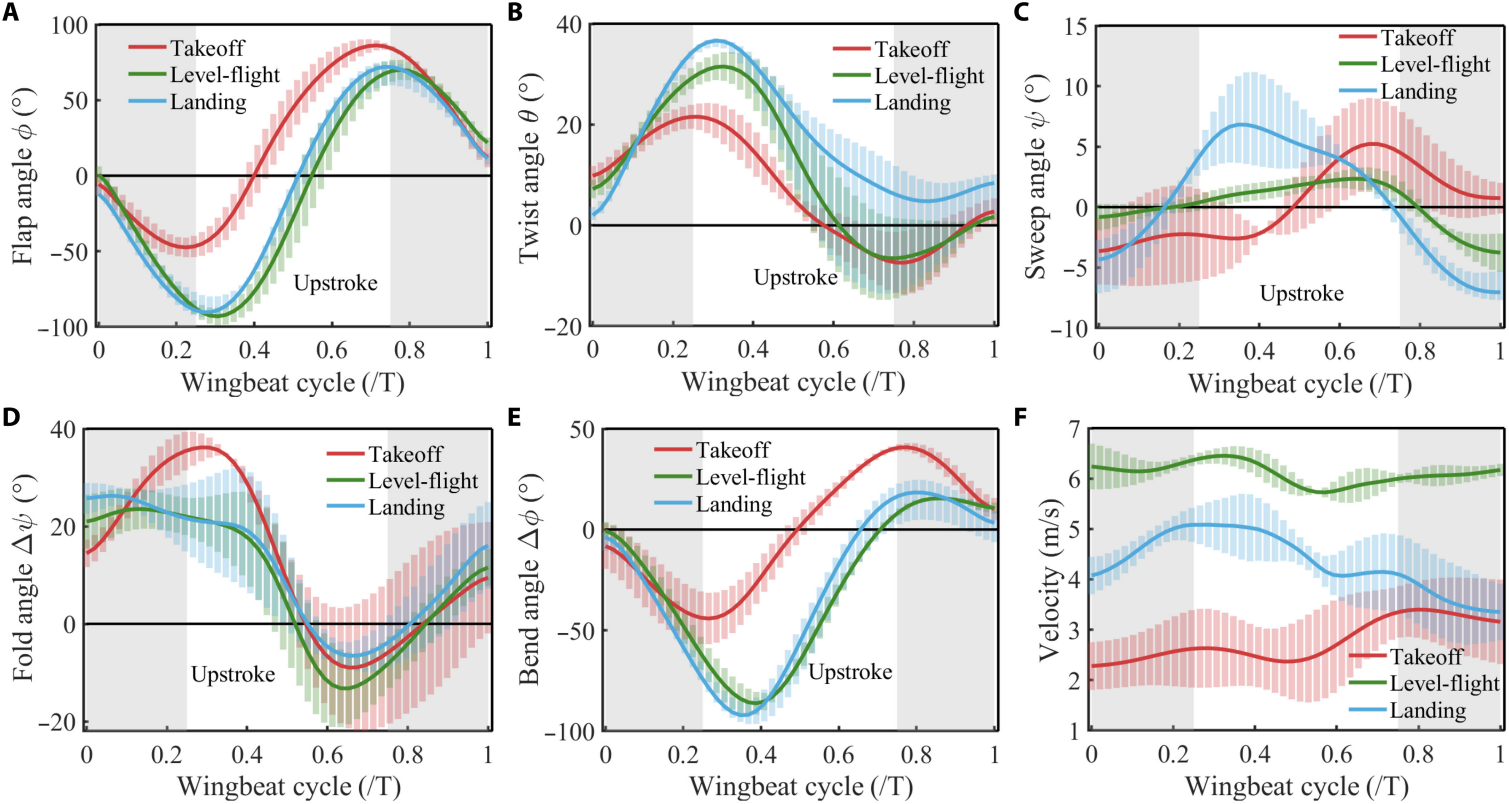
The differentiation of angles and velocity is in 3 stages. Red indicates the takeoff stage, green indicates the leveling flight stage, and blue indicates the landing stage. The average values of 5 pigeons in the experiment are taken. The error bars represent the angles observed in the 5 pigeons. (A) Differences in flap angles across stages. (B) Differences in twist angles across stages. (C) Differences in sweep angles across stages. (D) Differences in fold angles across stages. (E) Differences in bend angles across stages. (F) Flight velocity changes in different stages.

Fig. 6 shows the average angles and velocities for the 5 pigeon takeoff, leveling flight, and landing stages. Five sets of data were selected for each pigeon. Here, the angles are normalized to a period *T*. To clearly describe the upstroke and downstroke, we quantify these within a single wingbeat cycle, and the angular variations can be represented using first-order or higher-order Fourier functions. Here, $\hat{t}$ represents the dimensionless time within one wingbeat cycle, ranging from 0 to 1:

$$\begin{array}{c} \begin{array}{cc} \phi \left(\hat{t}\right)=\phi_{0}+\sum_{n=1}^{2}\left[\phi_{cn}\text{cos}\left(2\mathrm{n\pi}\hat{t}\right)+\phi_{sn}\text{cos}\left(2\mathrm{n\pi}\hat{t}\right)\right], & \\ \psi \left(\hat{t}\right)=\psi_{0}+\sum_{n=1}^{4}\left[\psi_{cn}\text{cos}\left(2\mathrm{n\pi}\hat{t}\right)+\psi_{sn}\text{cos}\left(2\mathrm{n\pi}\hat{t}\right)\right], & \\ \theta \left(\hat{t}\right)=\theta_{0}+\sum_{n=1}^{3}\left[\theta_{cn}\text{cos}\left(2\mathrm{n\pi}\hat{t}\right)+\theta_{sn}\text{cos}\left(2\mathrm{n\pi}\hat{t}\right)\right], & \\ \Delta \phi \left(\hat{t}\right)=\Delta \phi_{0}+\sum_{n=1}^{2}\left[\Delta \phi_{cn}\text{cos}\left(2\mathrm{n\pi}\hat{t}\right)+\Delta \phi_{sn}\text{cos}\left(2\mathrm{n\pi}\hat{t}\right)\right], & \\ \Delta \psi \left(\hat{t}\right)=\Delta \psi_{0}+\sum_{n=1}^{4}\left[\Delta \psi_{cn}\text{cos}\left(2\mathrm{n\pi}\hat{t}\right)+\Delta \psi_{sn}\text{cos}\left(2\mathrm{n\pi}\hat{t}\right)\right] & \end{array} \end{array}$$ (7)

Fig. 6A shows the flapping angles for the 3 stages, demonstrating a notable significant difference between different stages. In the takeoff stage, the downstroke angle is smaller, but the maximum angle during the upstroke is more important than other stages. In contrast, there is little difference between the leveling flight and landing stages.

Fig. 6B shows that during the takeoff stage, the wings have a smaller twist angle during the downstroke, and the overall twist magnitude is also relatively small. In contrast, the leveling flight and landing stages show more significant variations, with the twist angle in the landing stage remaining positive and differing significantly from the takeoff and leveling flight stages, especially in the late downstroke.

Fig. 6C shows that the sweep angle magnitude changes little within a single wingbeat cycle of 3 stages. However, each flight state exhibits different angular variation trends, with the sweep angle in the takeoff stage showing a generally slow increase, thus driving the wing movement in the positive $X_{s}$ direction, while the leveling flight phase remains stable throughout the flapping cycle and then experiences a sudden decrease during the final downstroke. The landing stage shows the most pronounced changes, with an apparent increase in angle during the mid-downstroke and mid-upstroke, followed by a continuous decrease after that.

Fig. 6D shows the fold angle. The overall fold angles for leveling flight and landing stages are similar, whereas the takeoff stage differs in the early part of the downstroke and upstroke. The angle changes in the leveling flight and landing stages are relatively smooth, whereas the takeoff stage shows a significant increase and decrease in angle.

Fig. 6E illustrates the bend angles similar to the flapping angles. It is evident that throughout the wingbeat cycle, the magnitude of change in the bend angle during the takeoff stage is markedly different from that observed in the other 2 stages. In the takeoff stage, the bend angle changes less during the downstroke and reaches a larger maximum during the upstroke, with similar patterns seen in the leveling flight and landing stages.

Fig. 6F represents the velocity changes for the 3 stages within one cycle. It shows that during the takeoff stage, the pigeon’s speed gradually increases, whereas during leveling flight, the speed remains relatively stable and higher, and during landing, the speed decreases gradually. The average speeds are ${\bar{v}}_{\text{takeoff}}=2.77\;m/s$ for takeoff, ${\bar{v}}_{\text{flight}}=6.10\;m/s$ for leveling flight, and ${\bar{v}}_{\text{landing}}=4.34\;m/s$ for landing, and these average speeds are used as the inflow velocities *U* in the CFD simulations.

### Aerodynamic analysis

**Fig. 7.**
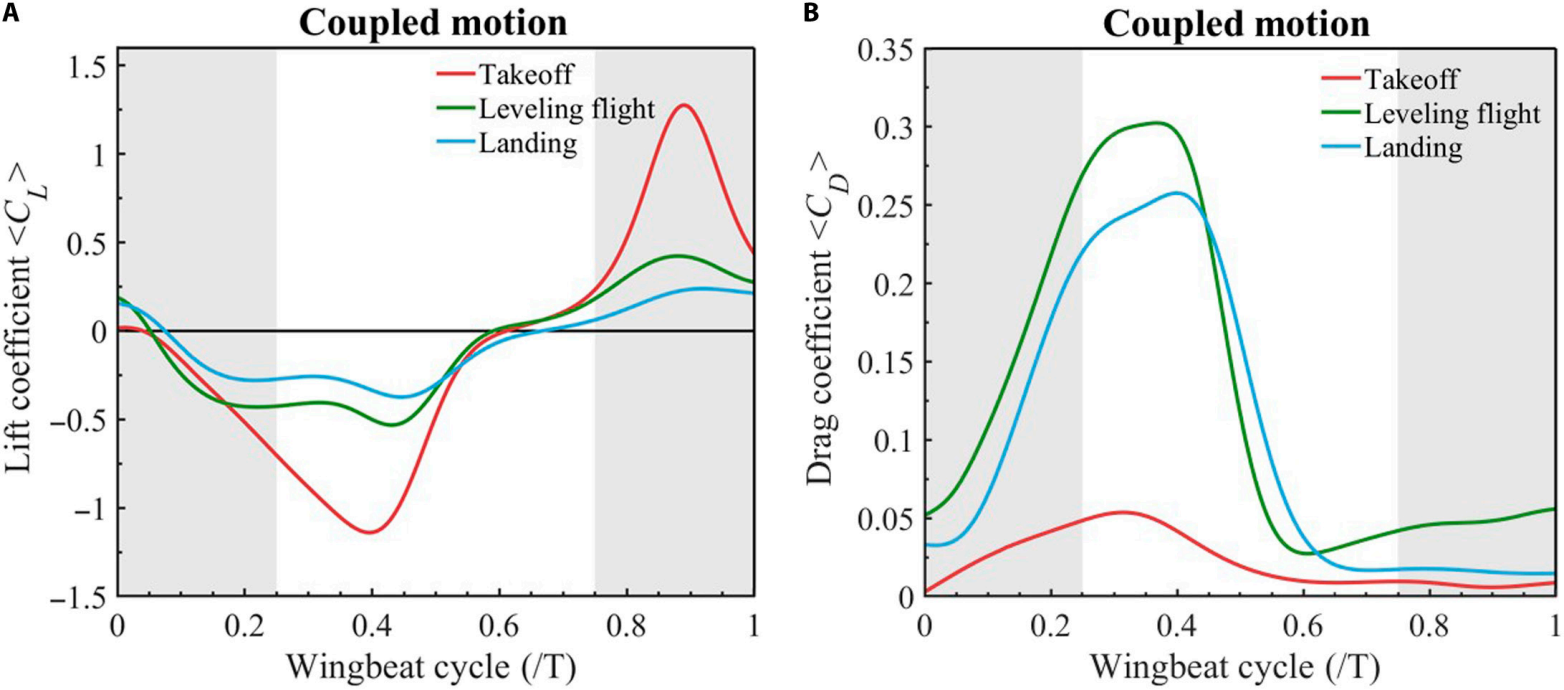
Comparison of force coefficients between 3 stages. Red indicates the takeoff stage, green indicates the leveling flight stage, and blue indicates the landing stages. (A) Comparison of lift coefficients. (B) Comparison of drag coefficients.

The aerodynamic results are divided into 3 parts: takeoff, leveling flight, and landing. This section primarily analyzes the aerodynamic forces of the wing. Fig. 7 shows the force coefficients during the takeoff, leveling flight, and landing stages, clearly indicating significant differences in lift and drag coefficients across different flight stages.

Fig. 7A details the 3 stages’ lift coefficient distribution within one wingbeat cycle. The lift coefficient during the takeoff stage is significantly higher than that during the other 2 stages, with a negative lift peak at 0.4 T. During the subsequent upstroke phase, the lift continues to increase, with the amplitude variation being noticeably more significant than during the leveling flight and landing stages. The lift peaks at 0.9 T and rapidly decreases during the subsequent downstroke phase. The significant fluctuations in peak lift demonstrate that the pigeon’s flight state during the takeoff stage is not yet stable, requiring substantial energy to overcome gravity. Fig. 7B shows the 3 stages’ drag coefficient distribution within one wingbeat cycle. Similarly, the drag coefficient during the takeoff stage is significantly lower than during the other 2 stages, especially during the first half of the downstroke and the early phase of the upstroke. Although the drag coefficient peaks at 0.3 T, the amplitude variation remains small. Therefore, in the early and middle phases of the takeoff stage, the pigeon generates a significant negative lift to assist in takeoff. In the subsequent process, lift rapidly increases to overcome gravity while maintaining low drag to achieve sufficient thrust for forward flight.

Fig. 7A shows that during the leveling flight stage, the lift coefficient changes steadily in the early and late stages of flight, with minimal fluctuations and no significant peaks. The amplitude is relatively symmetrical, fluctuating from −0.5 to 0.5. However, as shown in Fig. 7B, the drag coefficient during the leveling flight stage exhibits significant variation during the flight cycle, particularly in the early part of the downstroke, where the drag coefficient increases markedly. From 0.25 to 0.4 T in the upstroke, the drag coefficient remains stable before rapidly decreasing and reaching a low point at 0.55 T. The subsequent upstroke and downstroke phases are relatively stable. This indicates that the pigeon’s wingbeat generates sufficient lift during leveling flight to maintain flight speed and balance. As illustrated in Fig. 6F, the pigeon’s flight speed remains relatively stable throughout this process, but it requires managing considerable drag to sustain flight speed.

During the landing stage, as shown in Fig. 7A, the lift remains within a relatively small range of variation, and this demonstrates that the flight state is very stable at this time, with stable changes fluctuating between −0.25 and 0.25. However, during the landing stage, the pigeon’s drag coefficient rapidly increases within 0 to 0.4 T and stabilizes after decreasing to a lower value during the subsequent upstroke. Fig. 6F indicates that the pigeon’s speed drops sharply after 0.4 T during the descent process. During the landing phase, the pigeon gradually reduces its lift and increases drag to control its descent speed, achieving appropriate landing conditions.

Analysis of the results in this section indicates that during the takeoff phase, the pigeon seeks to maximize lift and minimize drag to achieve optimal forward flight conditions. In the leveling flight phase, it remains relatively stable. In the landing phase, lift is reduced as much as possible while drag is increased to achieve a smooth landing effect.

### Flow structure

**Fig. 8.**
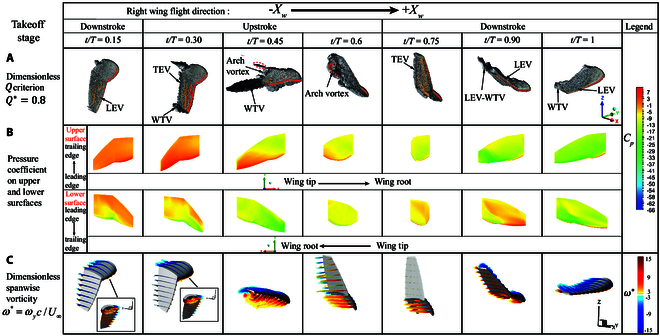
Flow structures during the takeoff stage. (A) *Q* criterion vortex structures throughout the wingbeat cycle, $Q^{*}=0.8$. (B) Pressure coefficient distribution on the upper and lower surfaces. (C) Spanwise vorticity $\omega^{*}=\omega_{y}c/{\bar{U}}_{\infty }$.

**Fig. 9.**
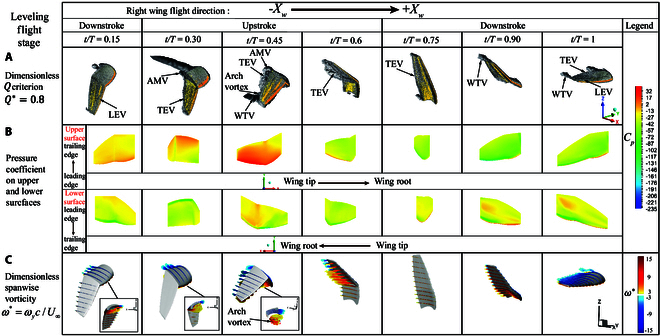
Flow structures during the leveling flight stage. (A) *Q* criterion vortex structures throughout the wingbeat cycle, $Q^{*}=0.8$. (B) Pressure coefficient distribution on the upper and lower surfaces. (C) Spanwise vorticity $\omega^{*}=\omega_{y}c/{\bar{U}}_{\infty }$.

**Fig. 10.**
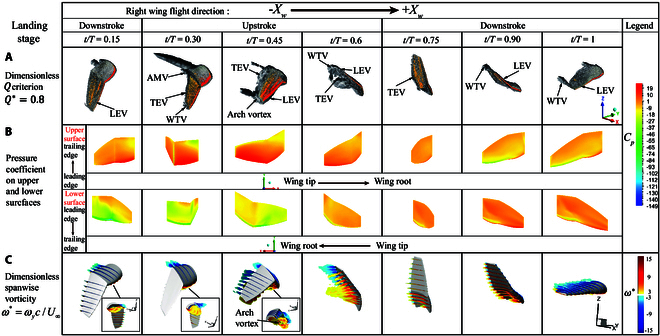
Flow structures during the landing stage. (A) *Q* criterion vortex structures throughout the wingbeat cycle, $Q^{*}=0.8$. (B) Pressure coefficient distribution on the upper and lower surfaces. (C) Spanwise vorticity $\omega^{*}=\omega_{y}c/{\bar{U}}_{\infty }$.

To further explain the changes at various moments within the flapping cycle of the pigeon, we observed and analyzed the fluid structure. To better observe the vortex structures generated by the coupled wing motion, this study visualized the vortices using the isosurface *Q* criterion and nondimensionalized *Q*, represented as $Q^{*}$:

$$\begin{array}{cc} Q=\frac{1}{2}\left({\left\|\omega \right\|}^{2}-{\left\|S\right\|}^{2}\right), & \\ Q^{*}=Q/{\left({\bar{U}}_{\infty }/\bar{c}\right)}^{2} & \end{array}$$ (8)

where ω and *S* represent the vorticity tensor and strain rate tensor, ${\bar{U}}_{\infty }$ is the average inflow velocity, and $\bar{c}$ is the average chord length. Figs. 8 to 10A illustrate the nondimensional isosurface *Q* criterion during the takeoff, landing, and leveling flight stages, with $Q^{*}=0.8$. Moreover, Figs. 8 to 10B provide detailed pressure distribution on the upper and lower surfaces of the wing throughout an entire wingbeat cycle. Additionally, Figs. 8 to 10B detail the pressure distribution on the upper and lower surfaces of the wing. To further understand vortex strength, we also studied the sectional cut of the nondimensional spanwise vorticity. The spanwise nondimensional coefficient $\omega^{*}$ is expressed as $\omega^{*}=\omega_{y}c/{\bar{U}}_{\infty }$, and the results are shown in Figs. 6 to 8C.

Takeoff stage: Fig. 8 shows the dimensionless *Q* criterion contour plot, pressure distribution on the upper and lower surfaces of the wing, and spanwise vorticity for the takeoff stage. At 0.15 T, the wing starts its downstroke motion from a fully extended position, with the leading-edge vortex (LEV) attaching to the upper and lower surfaces of the wing. At this moment, the fold angle of the hand wing begins to increase rapidly, and the sweep angle also gradually increases while the twist angle slowly increases. This creates a small region of low pressure at the leading edge of the junction between the hand wing and arm wing. By 0.3 T, as the hand wing and arm wing move upwards, the low-pressure region on the lower surface gradually moves toward the trailing edge and wing tip, forming a clear wing tip vortex at the tip. The hand wing exhibits a noticeable trailing-edge vortex (TEV), while a substantial amount of vorticity gathers beneath the arm wing, as depicted in Fig. 8C. As the wing moves upward, the twist angle decreases, and the fold angle rapidly decreases. Therefore, at 0.45 T, a large amount of vorticity accumulates toward the wing tip, making the wing-tip vortex (WTV) more prominent. Due to the decrease in the twist angle, an arch vortex forms on the wing’s lower surface. Additionally, the difference in wingbeat angles between the inner and outer wings causes some TEV to shed at the junction of the 2 wing sections. Consequently, during the takeoff stage, the first negative peak of the lift coefficient occurs around 0.4 T. At the same time, the pressure on the wing’s lower surface decreases overall, with the primary low-pressure region near the wing tip. At this point, the drag also reaches its maximum value. As the upward flap angle increases, between 0.45 and 0.6 T, the fold angle decreases. The arch vortex on the wing’s lower surface moves toward the wing tip, and TEV is shed from the hand wing’s trailing edge. As shown in Fig. 8C, this leads to a significant increase in lift during this period. Subsequently, at 0.75 T, the twist angle increases while the sweep angle decreases. During this process, the arch vortex gradually moves toward the wing tip, and vortices shed at the junction of the hand wing and arm wing. During the downstroke phase from 0.75 to 1 T, the fold angle increases, and the LEV gradually moves toward the wing tip. The spanwise vorticity plot also shows stronger vortices near the wing tip. At 0.9 T, a distinct WTV forms. With a slight increase in the twist angle and an increase in the downward flap angle, high-pressure regions form near the trailing edge on the upper surface and the wing tip on the lower surface, leading to maximum lift. As the process continues to 1T, the LEV moves toward the wing tip. Fig. 8C shows that the vortex structures near the wing tip become more prominent, and the wing tip vortex sheds between 0 and 0.15 T in the next wingbeat cycle.

Leveling flight stage: Fig. 9 presents the dimensionless contour plots of the *Q* criterion, pressure distribution on the upper and lower surfaces of the wing, and spanwise vorticity section during the leveling flight stage. From 0 to 0.15 T, the wing rapidly flaps downward. During this period, the angle changes during leveling flight are more significant than during takeoff. The fold angle remains around 20° while the twist angle begins to increase. During this phase, the LEV remains attached, and the WTV sheds, with a substantial amount of vorticity accumulating beneath the hand wing, as shown in Fig. 9C. From 0.15 to 0.3 T, the wing completes the downstroke and begins the upstroke. Due to the angle difference between the hand wing and arm wing, an arm wing vortex (AMV) forms at the junction, and more vorticity accumulates in the hand wing section. The LEV has not yet formed on the upper surface, resulting in a low-pressure region on the lower surface. As the hand wing continues its upward flap, the twist and fold angles gradually decrease. By 0.45 T, a prominent WTV forms, and the AMV sheds. The vorticity on the lower surface of the hand wing and LEV on the lower surface of the arm wing gather, forming an arch vortex with the TEV, which aids in generating flight thrust. The lift coefficient and drag coefficient reach their peak near this point. Between 0.45 and 0.6 T, the WTV and AMV continue to shed, resulting in a rapid increase in lift and a decrease in drag. The fold angle reaches its minimum and increases, while the sweep angle reaches its maximum and starts to decrease. Consequently, toward the end of the upstroke, the vortices shed more quickly, and due to the large flap angle, the projected area of the wing in the direction of the flow reaches its minimum. The downstroke begins between 0.75 and 0.9 T. Due to increases in the twist and fold angles, a high-pressure region forms at the leading edge of the lower surface, causing the LEV to reattach quickly and the WTV to reform at the wing tip. During the downstroke, a slight increase in the twist angle and a rapid increase in the fold angle result in the LEV concentrating toward the WTV, which helps in shedding the WTV from 0.9 to 1 T, thus improving thrust.

Landing stage: Fig. 10 illustrates the dimensionless *Q* criterion contour plot, pressure distribution on the upper and lower surfaces of the wing, and spanwise vorticity section plot for the landing stage. During the early part of the wingbeat cycle, the flap, fold, and bend angles in the landing stage are similar to those during the leveling flight. However, the twist angle rapidly increases from the beginning of the wingbeat, resulting in a similar variation from 0 to 0.15 T as observed in leveling flight. During this period, the WTV is shed while the LEV remains stable, and a significant amount of vortices accumulates beneath the hand wing, as shown in Fig. 10C. From 0.15 to 0.3 T, with the rapid increase in twist and sweep angles, the LEV gradually shifts toward the trailing edge of the wing, and an AMV forms at the boundary between the hand wing and arm wing due to the angle differences. During the upstroke from 0.3 to 0.45 T, the AMV begins to shed from the boundary, and due to the reduction in the twist angle, the LEV on the lower surface accumulates toward the wingtip, forming an arch vortex and enhancing the WTV formation. A substantial amount of vortices moves toward the wing’s trailing edge, and a significant low-pressure region forms at the trailing edge of the hand wing, as observed in Fig. 10C. From 0.45 to 0.6 T, the rapid decrease in the fold angle leads to the shedding of the WTV and boundary vortices, causing a gradual increase in lift until 0.75 T when the WTV is completely shed, marking the end of the upstroke. During the downstroke, the twist angle remains upbeat with minimal changes, and the LEV reattaches, generating a new WTV due to the downward motion toward the wingtip. A small low-pressure region forms near the wingtip on the upper surface. In the following period, the sweep angle slightly decreases, and the fold angle increases. In contrast, the twist angle remains relatively stable, causing the LEV to keep moving toward the wingtip without the shedding of the WTV.

The observations and analysis above indicate apparent differences in flow structures caused by wing morphing across different flight stages. During takeoff, the pigeon employs rapid downstrokes and minor twist angle adjustments to achieve more excellent lift and makes substantial fold angle changes to maintain stability. In the leveling flight stage, the pigeon balances its weight and enhances forward thrust by deforming its wings while sustaining lift. For landing, the pigeon increases drag to lower its speed and maintain posture while keeping lift, ensuring a stable landing.

## Discussion

Birds flexibly morph their wings during takeoff, leveling flight, and landing to achieve optimal flight performance for each stage. This study investigates pigeon wings’ changes and aerodynamic characteristics in these 3 flight stages. We collected kinematic information on the wings during free flight. We thoroughly analyzed the kinematic data for takeoff, leveling flight, and landing, categorizing them into 5 specific parameters: wingbeat, twist, sweep, fold, and bend. We also obtained detailed velocity changes within a wingbeat cycle. We analyzed the vortex structures, pressure distributions on the upper and lower surfaces, and spanwise vorticity sections during these stages using CFD simulations. The study further examines how changes in kinematic angles influence the flow field structure to achieve the superior aerodynamic performance required for each flight stage.

Our biological experiments and simulation results demonstrate that each kinematic parameter varies significantly across different flight stages. These significant variations lead to changes in flow field structure, thereby causing the distinct aerodynamic performances observed at different flight stages. Compared to the other 2 stages, the differences in angle changes within the stroke plane between the arm and hand wings are more minor during takeoff. As a result, vortex separation and formation primarily occur in the hand wing. During the initial stage of the wingbeat cycle, from 0 to 0.3 T, the 2 angles within the stroke plane begin their upward motion earlier. Simultaneously, the increase in the fold angle causes the hand wing to align more with the incoming flow. As a result, the WTV forms earlier than the other 2 stages. However, due to a smaller twist angle, the vortex on the lower surface converges toward the wingtip more quickly than the upper surface, and no AMV forms at the junction between the arm wing and the hand wing. Consequently, this generates more negative lift during the takeoff stage, and the pigeon’s drag gradually increases to its maximum. Thus, in the early stages of the wingbeat cycle, the pigeon must adjust its posture and gradually increase its speed. The steady posture makes lift variations more stable during the takeoff and landing phases. The wing has undergone significant changes in the twist angle, and there are also large differences in angles within the stroke plane. These changes lead to a substantial vortex accumulation near the wing’s lower surface, forming the AMV. As a result, drag increases rapidly during this process.

During the subsequent upstroke, the LEV rapidly attaches to the takeoff stage due to the reduction in twist and fold angles. The slight angle differences within the stroke plane cause the vortex structure from the arm wing to migrate toward the hand wing, eventually forming an arch vortex. Furthermore, the continuous reduction in the fold angle up to 0.7 T leads to the persistent formation of the WTV, resulting in a sustained increase in the lift coefficient throughout this process. In contrast, this process is relatively delayed during the leveling flight and landing stages. The larger twist angle prevents the LEV from attaching well, leading to continuous shedding of AMV and TEV. As a result, changes in lift are less evident and show some fluctuations, especially during the landing stage. This shedding of vortices causes drag to decrease sharply during these 2 stages. After 0.5 T, with the decrease in twist angle and the gradual increase in fold angle, the LEV reattaches in the leveling flight and landing stages, causing the lift to increase gradually. However, during the landing stage, the limited changes in fold and twist angles lead to significant vortex accumulation in the hand wing by 0.6 T during the upstroke, resulting in a less noticeable increase in lift during this period.

The 3 stages show no significant differences as the upstroke ends and the downstroke begins. The LEV reattaches and moves toward the wingtip, forming the WTV and creating a low-pressure region near the wingtip. During the downstroke from 0.75 to 1 T in the takeoff and leveling flight stages, the twist angle increases again, causing the WTV to shed. This results in a slight lift decrease and a slight drag increase after 0.9 T. During the landing stage, there is no significant change in the twist angle of around 0.9 T to maintain body posture stability. As a result, the lift remains stable during this process.

During the 3 stages of flight, the definition of wing movement has the greatest impact on aerodynamic performance. The flapping motion is the basic motion in the coupled motion during the pigeon wing beat cycle. It allows the pigeon to maintain its gravity in forward flight and provides the most basic lift and thrust. The flapping and bending motions, both in the stroke plane, play a similar role in influencing aerodynamic performance. However, the bending motion effectively reduces the area of the wing in the vertical projection plane. The folding motion can also change the wing area. Sweeping and folding motions outside the stroke plane affect the timing of the LEV attachment. However, the aerodynamic effect is minimized due to the small angle of the sweeping motion. The most significant influence on lift and drag is the twisting motion, which changes the upwind surface of the wing and most effectively affects the effective AOA of the whole wing. However, since this article analyzes the fully coupled motions, the specific effects of each motion on the force coefficients need to be specifically analyzed in future work.

Medium-sized birds have more complex kinematic transformations during flight. Thus, decoupling and classifying the kinematics is necessary, unlike previous studies on other flyers with flapping flight [27–29]. This method improves our understanding of the kinematic mechanisms at each flight stage and enables a more precise analysis of how different angle couplings affect aerodynamic performance. Unlike research on bio-inspired flapping-wing vehicles [30–32], we deviate from simple periodic motion patterns by basing our study on biological data. Our work enables us to use the true capabilities of exceptional natural fliers as a genuine guide for biomimetics. This method also applies to future studies involving similar biological or engineering research.

Studying the kinematics and aerodynamics of complex coupled motions during different flight stages enables us to understand better why birds choose such motions at these stages: (a) A deep understanding of the wing motion patterns at different flight stages, decoupled into parameters familiar with robot design and incorporating biomimetic wing designs, provides data support and guidance for multi-degree-of-freedom wing configurations in flapping wing robots. This can guide the design of efficient drive systems, ensuring that the power output of the flapping wings can respond in real time to adjustments in control strategies. (b) The deformation choices of birds during takeoff and landing stages, dynamic adjustments of the wingbeat cycle, and control of vortex generation and shedding can inform the control strategies of flapping wing robots to adapt to environmental changes and improve performance at different flight stages. (c) Research into aerodynamic mechanisms guides the design of robot models and helps fill gaps in the reinforcement learning flight datasets for flapping wing robots, enhancing simulation and real-world datasets. By simulating potential external disturbances, the generalization ability of reinforcement learning models can be improved [33–35].

It is important to note that while this study extensively investigates coupled CFD simulations at different flight stages, we have treated the wings as rigid bodies, and the twist angle observed in actual flight could be a result of feather deformation caused by air pressure, rather than a completely voluntary twist by the pigeon. Thus, in reality, birds possess more flexible wing deformations due to feathers, which enhance their flight performance [36,37]. Hence, future research should incorporate fluid–structure interaction considerations. Furthermore, the study reveals that substantial variations of kinematic parameters significantly impact aerodynamic performance in coupled motions. However, existing flapping-wing aerial vehicles (FWAVs) mostly have single modes of wing motion, making it difficult to achieve complex and highly coupled motions like those of birds. Therefore, future work should prioritize decoupling these motions, simulating each parameter separately, or comparing the effects of various kinematic parameter couplings.

## Data Availability

All data needed to evaluate the conclusions in the paper are presented in the paper.
